# Design and rationale for a comparison study of Olmesartan and Valsartan On myocardial metabolism In patients with Dilated cardiomyopathy (OVOID) trial: study protocol for a randomized controlled trial

**DOI:** 10.1186/s13063-021-05970-7

**Published:** 2022-01-15

**Authors:** Sua Jo, Hyeyeon Moon, Kyungil Park, Chang-Bae Sohn, Jeonghwan Kim, Yong-Seop Kwon, Su Hong Kim

**Affiliations:** 1 Regional Cardiocerebrovascular Center, Dong-A University Hospital, Division of Cardiology, Department of Internal Medicine, Dong-A University College of Medicine, Daesingongwon 26, Seo-gu, Busan, 49201 Republic of Korea; 2 Dong-A University College of Medicine, Busan, Republic of Korea; 3 Ulsan Medical Center, Ulsan, Republic of Korea; 4 Dong-eui Hospital, Busan, Republic of Korea; 5 Busan St. Mary’s Hospital, Busan, Republic of Korea; 6 Busan Veterans Hospital, Busan, Republic of Korea

**Keywords:** Olmesartan, Valsartan, Myocardial metabolism, Positron emission tomography

## Abstract

**Background:**

Dilated cardiomyopathy (DCMP) is characterized by ventricular chamber enlargement and systolic dysfunction which may cause heart failure. Patients with DCMP have overactivation of the renin-angiotensin-aldosterone systems, which can also adversely affect myocardial metabolism in heart failure. The impairment of myocardial metabolism can contribute to the progression of left ventricular remodeling and contractile dysfunction in heart failure. Although angiotensin II receptor blockers (ARBs) have been used to treat patients with DCMP, there has been no direct comparison of the efficacy of these agents. The objective of this study is to compare the effects of olmesartan and valsartan on myocardial metabolism in patients with DCMP.

**Methods/design:**

The OVOID study (a comparison study of Olmesartan and Valsartan On myocardial metabolism In patients with Dilated cardiomyopathy) is designed as a non-blinded, open-label, parallel-group, prospective, randomized, controlled, multicenter clinical trial. A total of 40 DCMP patients aged between 20 and 85 years will be randomly allocated into the olmesartan or the valsartan group. ^18^F-fluoro-2-deoxyglucose (FDG) cardiac positron emission tomography (PET) will be performed at baseline and six months after receiving the study agent. The primary endpoint is myocardial glucose consumption per square meter, measured using ^18^F-FDG PET 6 months after receiving the study agent.

**Discussion:**

The purpose of this trial is to compare the efficacy between olmesartan and valsartan in improving myocardial metabolism in DCMP patients. This will be the first randomized comparative study investigating the differential effects of ARBs on heart failure.

**Trial registration:**

ClinicalTrials.govNCT04174456. Registered on 18 November 2019

**Supplementary Information:**

The online version contains supplementary material available at 10.1186/s13063-021-05970-7.

## Background

Dilated cardiomyopathy (DCMP) is characterized by ventricular chamber enlargement and systolic dysfunction which may cause heart failure (HF) [1]. Increased wall stress and oxygen demand result in increased myocardial oxygen consumption in DCMP [2]. At the same time, myocardial energy efficiency is reduced with wasteful cycling of free fatty acids through lipolysis, re-esterification, and suppression of the more energy-efficient glucose metabolism [3, 4]. Thus, patients with DCMP may exhibit alterations in myocardial metabolism. Recent studies have suggested that the suppression of the renin-angiotensin-aldosterone systems (RAAS) might have potential myocardial metabolism benefits in DCMP patients [5, 6]; overactivation of the RAAS is one of the key detrimental mechanisms of DCMP progression and is associated with poor prognosis [7, 8]. The increased adrenergic tone in HF (1) not only exerts a direct toxic effect on myocytes (2) but also causes unfavorable changes in myocardial energy use [9, 10]. Therefore, suppression of RAAS could positively affect cardiac energy metabolism and is a potential therapeutic strategy in patients with DCMP. Angiotensin II receptor blockers (ARBs) have been shown to be effective in RAAS suppression [11], and currently, several ARBs have been approved for clinical use. However, there has been no direct comparison of the efficacy of different ARBs in HF patients. A previous study demonstrated that treatment with olmesartan significantly improved left ventricular (LV) function and ameliorated the progression of cardiac remodeling in rats with DCMP [12]. It has been reported that the antihypertensive action and duration of olmesartan may be greater than those of other ARBs because it is a more potent and selective angiotensin II receptor antagonist with no agonist activity [13–17].

The purpose of this study was to noninvasively compare the effects of olmesartan vs. valsartan on myocardial metabolism in nonischemic myocardial segments of patients with DCMP.

## Objectives

### Primary objective

The primary objective is to compare the effects of 6-month olmesartan and valsartan treatment on myocardial metabolism in DCMP patients.

### Secondary objectives

The secondary objectives are to compare the effects of 6-month olmesartan and valsartan treatment in DCMP patients on the following variables: (1) change in myocardial glucose consumption, (2) change in N-terminal pro-B-type natriuretic peptide (NT-proBNP) level, (3) change in left ventricular ejection fraction (LVEF), (4) change in New York Heart Association (NYHA) functional class, and (5) the occurrence of clinical events.

## Methods/design

### Participants

Patients are eligible for this study if they are diagnosed with DCMP with an LV ejection fraction of < 40% and an LV end-diastolic diameter of > 117% of the predicted value corrected to body surface area and age with NYHA functional classes of III and IV (Table 1). A diagnosis of DCMP is determined using the currently accepted criteria [18].

**Table 1:** Inclusion and exclusion criteria

Inclusion criteria	
1) Patients with a diagnosis of heart failure NYHA functional class III or IV 2) Left ventricular end-diastolic diameter greater than 117% of the predicted value corrected for body surface area and age 3) Left ventricular ejection fraction ≤ 40% 4) Not planned for revascularization 5) Absence of severe intractable arrhythmias	
Exclusion criteria	
1) Less than 20 years or more than 85 years old 2) The presence of hemodynamic instability 3) Known intolerance to olmesartan and valsartan 4) Coronary artery disease 5) Acute or subacute stage of myocarditis 6) Primary valve disease 7) Excessive use of alcohol 8) Expected or performed cardiac resynchronization therapy and heart transplantation 9) Stress-provoked Takotsubo cardiomyopathy 10) Tachycardia-induced cardiomyopathy 11) Peripartum cardiomyopathy 12) Cor pulmonale 13) Impaired renal function 14) A life expectancy < 1 year 15) An inability to give informed consent.	

*NYHA* New York Heart Association

The exclusion criteria are as follows: (1) less than 20 years and more than 85 years old, (2) the presence of hemodynamic instability, (3) known intolerance to olmesartan and valsartan, (4) coronary artery disease based on coronary angiography (≥ 50% stenosis in ≥ 1 of the major coronary arteries) and/or a history of myocardial infarction or angina pectoris, (5) acute or subacute stage of myocarditis, (6) primary valve disease, (7) excessive use of alcohol, (8) expected or performed cardiac resynchronization therapy and heart transplantation, (9) stress-provoked Takotsubo cardiomyopathy, (10) tachycardia-induced cardiomyopathy, (11) peripartum cardiomyopathy, (12) Cor pulmonale, (13) impaired renal function (estimated glomerular filtration rate of < 60 ml/min/1.73m^2^, (14) a life expectancy of less than 1 year, and (15) inability to provide informed consent. The complete lists of inclusion and exclusion criteria are provided in Table 1. Patients will not be excluded from the analysis after randomization. The participants will be dropped out of the study if (1) the subjects choose to withdraw from the study, (2) there is poor treatment compliance, (3) they experience serious adverse events or present a serious laboratory abnormality that constitutes an unacceptable risk with continued participation in the study, and (4) they are pregnant.

### Study design

**Fig. 1:**
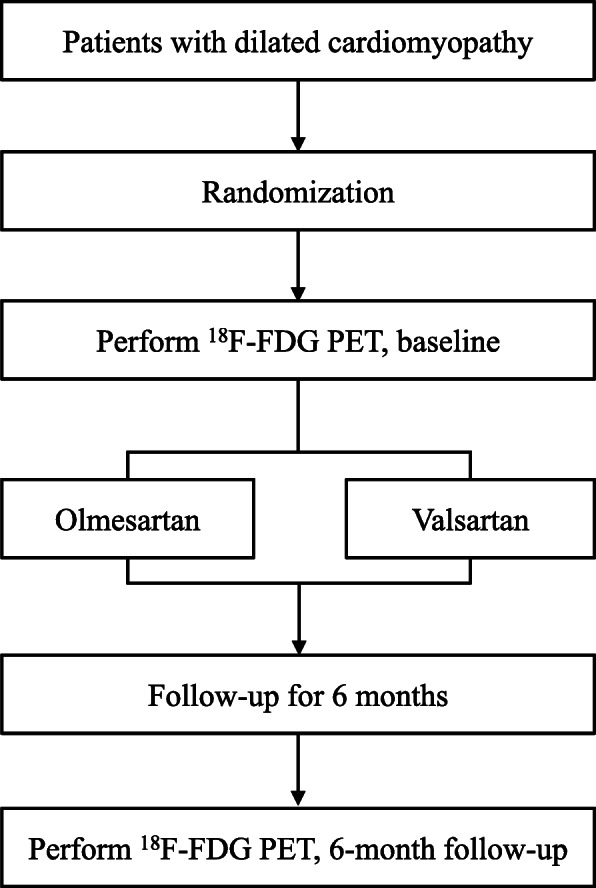
Study design. FDG, fluoro-2-deoxyglucose; PET, positron emission tomography

OVOID study (a comparison study of Olmesartan and Valsartan effects on myocardial metabolism in patients with Dilated cardiomyopathy) is a prospective, multicenter, randomized, open-label, active-controlled study with two parallel study groups. The study is being conducted in five tertiary hospitals in South Korea. The participants are randomly allocated into the olmesartan group and the valsartan group and followed-up for 6 months after discharge. The overall study algorithm is depicted in Fig. 1. A diagnostic coronary angiogram is performed on all patients to exclude coronary artery disease. If not contraindicated, the patients receive evidence-based pharmacological therapy (at the maximum tolerated dosages), except angiotensin-converting enzyme (ACE) inhibitor and ARB [19]. A baseline echocardiographic examination and ^18^F-fluoro-2-deoxyglucose (FDG) PET scan are performed before discharge. During the study period, study visits are scheduled at 1, 3, and 6 months. At each visit, the patients undergo a complete physical examination, medical history collection, and assessment of drug compliance. At each study visit, a sample of blood is sent to a central laboratory for the measurement of plasma NT-proBNP. The investigators evaluate all clinical and laboratory adverse events at each visit. To monitor safety, serum creatinine and blood urea nitrogen concentrations are determined at every study visit. The patient’s NYHA functional class and predefined clinical events are recorded at each clinical visit. ^18^F-FDG PET and echocardiography will be performed 6 months after randomization [18] by a core laboratory staff blinded to random assignment. Table 2 summarizes the study enrollment, interventions, and assessments details.

**Table 2:** Participant timeline

Time point	Study period								
	Enrolment	Olmesartan group				Valsartan group			
Time	Week 0	Week 1	Week 4	Week 12	Week 24	Week 1	Week 4	Week 12	Week 24
**Enrolment**									
Eligibility screen	**○**								
Informed consent	**○**								
**Interventions**									
OVOID 24 weeks program		**○**	**○**	**○**	**○**	**○**	**○**	**○**	**○**
**Assessments**									
Demographic data	**○**								
Baseline PET	**○**								
6-month PET					**○**				**○**
Secondary outcomes					**○**				**○**
Clinical examination	**○**	**○**	**○**	**○**	**○**	**○**	**○**	**○**	**○**
Adverse events		**○**	**○**	**○**	**○**	**○**	**○**	**○**	**○**

*PET* Positron emission tomography

The study protocol (version # 2.3, 6 November, 2019), which follows the SPRIT guidelines (see the additional file, appendix A), was approved by the Institutional Review Board. The recruitment of participants started in December 2019 and was completed in December 2021. This study follows the principles of the Declaration of Helsinki, whereby all patients sign a written informed consent stating that participation is voluntary and could be withdrawn at any time without any negative consequences concerning their current or future medical treatment. Written informed consent is obtained from each participant before ^18^F-FDG PET and randomization. The trial is overseen by an independent data monitoring committee and registered at www.clinicaltrials.gov: NCT04174456.

### Study endpoints

The primary hypothesis to be tested is the efficacy of olmesartan in significantly improving myocardial metabolism in DCMP patients compared to valsartan with a 6-month follow-up period. Myocardial metabolism is visualized using PET with metabolic tracers; this study uses PET and ^18^F-FDG to assess myocardial glucose use. The primary endpoint is myocardial glucose consumption measured by PET 6 months after randomization. The secondary endpoints include changes in myocardial glucose consumption, NT-proBNP levels, LVEF, NYHA functional class from baseline to the last available observation after treatment, and the occurrence of predefined clinical events after receiving the study agent.

In this study, clinical events are all causes of death, cardiovascular death, left ventricular assist device implantation, listing for cardiac transplantation, hospitalization for worsening HF, and intensification of therapy defined by an increase in the diuretic dose of > 50% in the outpatient setting during the 6 months of follow-up. The adverse events will be reported from in-hospital observation and follow-ups. The study population will be instructed to contact the research staff via mobile phone at any time if adverse events occurred during the study period.

### Intervention and comparator descriptions

The eligible patients are randomly assigned to either the olmesartan or valsartan group in a 1:1 fashion. Study patients assigned to the olmesartan group take olmesartan 20 mg once daily. The control arm will receive valsartan because it is widely used to treat heart failure with reduced LVEF; valsartan 40 mg twice a day is administered, and then titrated to a target of 160 mg twice daily as tolerated throughout the study period [20]. If up-titration is not clinically feasible, either because of hypotension or deepening azotemia, the previous dose is subsequently administered as the maximal tolerable dose. Any patient who is found to take less than 80% or more than 120% of the assigned study drug as assessed by tablet counts is considered non-compliant [21].

## Assignment of interventions: allocation

### Sequence generation

Participants are randomly assigned to either the olmesartan or valsartan group in a 1:1 fashion. Randomization will take place following coronary angiography, which is conducted after stabilization by conservative treatment. Random assignments are generated using Excel spreadsheet software (Microsoft Corporation, Redmond, CA, USA).

### Concealment mechanism

Randomization is performed by a person that is not involved in this study and kept concealed. The connection will require a login, password, and study number which will be provided by the data manager from Dong-A University Hospital.

### Implementation

The assigned treatment will be administered for 6 months. We will take measures to minimize drop out and any potential biases attributable to dropout will be explored statistically. If a participant wishes to withdraw from the study, the reason for withdrawal will be documented in the participant records for the subsequent analysis in the interpretation of the results.

### ^18^F-FDG PET acquisition protocol and image interpretation

^18^F-FDG PET imaging is performed at the core lab (Dong-A University Hospital, Busan, South Korea). All studies are performed with a Biograph mCT flow scanner (Siemens Healthcare, Knoxville, TN, USA) consisting of a PET scanner coupled with a multi-detector CT scanner, allowing the acquisition of co-registered CT and PET images at the same time. After fasting for at least 6 h, different doses of insulin (1U-5U) are injected after 40–60 min of oral administration of 25–50 g of a 50% glucose load according to different blood glucose levels, for the patients to reach an optimal blood glucose concentration of 5.55–7.77 mmol/L [22]. After confirming that the proper blood sugar level is reached, low-dose CT is acquired, first with 100 kV, 30 mAs, 0.5 s each rotation, and 3.75 mm of slice thickness, without oral or intravenous contrast media before PET acquisition. Simultaneously with the initiation of the PET acquisition, 185 MBq of ^18^F-FDG is rapidly injected intravenously and the PET scan is acquired for 50 min in the list-mode. Standard static, 8-bin ECG-gated, and dynamic PET images are generated from the list-mode PET data, and the first two PET images (i.e., static and gated images) are reconstructed using the last 10 min of the entire data acquisition. Dynamic PET images are also reconstructed with 33-time frames (12 × 10 s, 3 × 20 s, 6 × 20 s, 4 × 60 s, and 8 × 5 min). These frames are generated with a 200 × 200 matrix, 2-mm slice thickness, a Gaussian filter of 4 mm in full width at half maximum, three iterations, and 21 subsets.

First, myocardial FDG uptake and functional status are evaluated visually and quantitatively using the QPS and QGS programs (Cedar-Sinai Medical Center, Los Angeles, CA, USA). To produce polar maps of the LV, reorientation and segmentation are performed in the dedicated program. Two experienced nuclear medicine physicians categorize the myocardial glucose uptake into four grades based on visual evaluation: grade 0 = minimal uptake, grade 1 = mostly minimal or mild uptake, grade 2 = mostly intense or moderate uptake, and grade 3 = homogeneously intense uptake [23]. When the grading is inconsistent between the two physicians, the grade is determined by mutual consultation.

Second, the dynamic PET data are analyzed using PMOD software (v3.6, Zürich, Switzerland) to assess myocardial glucose consumption. The PET image is automatically loaded using the PMOD Cardiac PET modeling (PCARDP) module to align the directions, and the volume of interest (VOI) is set by manually adjusting the detailed direction. To generate a time-activity-curve (TAC), VOIs of the LV, right ventricle (RV), and myocardium of the heart are generated. The selected PET image is smoothed with an FWHM 4 mm 3-dimensional Gaussian filter. It is confirmed that the RV TAC appears first, the LV TAC shows the next distinct peak, and finally, the myocardial TAC appears static. To perform the simulation, we select the ^18^F-FDG tracer and Patlak model from the PCARDP module. For the analysis of FDG data, the patient’s lumped constant (LC) and plasma glucose level (PG) are entered. Finally, the metabolic rate of glucose (MRGlu = slopexPG / LC) is obtained from the regression slope. For cardiac PET imaging, PCARDP uses the standard American Heart Association (AHA) 17-segment model. The final results are calculated in a table form and expressed in polar coordinates.

### Echocardiography

Echocardiographic examinations are performed at the participating centers and sent to the echocardiographic core lab (Dong-A University Hospital, Busan, South Korea). The analyses are performed by one experienced investigator. The biventricular dimensions and systolic and diastolic functions are assessed according to the international guidelines [24]. The left ventricular end-systolic diameter (LVESD) and left ventricular end-diastolic diameters (LVEDD) are measured by M-mode echocardiography. The LV volume and LVEF are calculated using a modified Simpson’s method. All volumes are indexed according to body surface area. All measurements are obtained from the mean of three beats (patients in sinus rhythm) or five beats (patients with atrial fibrillation).

## Cardiac ^123^I-metaiodobenzylguanidine scans and image analysis

The condition of cardiac sympathetic nerves is assessed using ^123^I-metaiodobenzylguanidine (MIBG), which is a norepinephrine analog and therefore is used as a tracer for sympathetic neuron integrity and function [25, 26], at baseline and 6 months after receiving the study agent. Early and delayed cardiac MIBG scans are performed at 30 min and 3 h after the intravenous injection of 111 MBq of ^123^I-MIBG, respectively. A planar image is obtained with a dual-head gamma camera equipped with a low-energy high-resolution parallel-hole collimator (eCAM, Siemens Healthcare, Knoxville, TN, USA). We also obtain a cardiac single-photon emission computed tomography (SPECT) image after acquiring the delayed planar image. Medications that could affect the MIBG uptake are withheld for 24 h prior to the administration of ^123^I- MIBG. Relative cardiac uptake is determined by setting the region-of-interest (ROI) on the anterior view. The entire left ventricular ROI is drawn manually, and a rectangular ROI is also set on the upper mediastinal area. The heart-to-mediastinum ratio (HMR) of the early (eHMR) and delayed (dHMR) images are calculated by dividing the average count density per pixel of the left ventricular ROI by that of the mediastinal ROI, respectively. A normal HMR is defined as greater than 1.8 in the delayed image, a number based on a previous study [27]. The wash-out rate (WR), which is an index of the rate at which MIBG is washed out between the early and the delayed images, is calculated using the following formula:

$$ \mathrm{WR}\;\left(\%\right)=\left[\left(\mathrm{early}\ \mathrm{cardiac}\ \mathrm{uptake}\hbox{-} \mathrm{early}\ \mathrm{mediastinal}\ \mathrm{uptake}\right)\hbox{-} \left(\mathrm{delayed}\ \mathrm{cardiac}\ \mathrm{uptake}\hbox{-} \mathrm{delayed}\ \mathrm{mediastinal}\ \mathrm{uptake}\right)\times 1.21\right]\times 100/\left(\mathrm{early}\ \mathrm{cardiac}\ \mathrm{uptake}\hbox{-} \mathrm{early}\ \mathrm{mediastinal}\kern0.17em \mathrm{uptake}\right) $$

A factor of 1.21 is multiplied by the delayed value to correct for the decay of ^123^I at 3 h [28].

### Adverse events

The analysis of safety-related data is performed with respect to the frequency of serious adverse events, stratified by the causality and intensity of the morbidity in both treatment groups. At each visit, the patients are interviewed about the occurrence of any adverse events, including the time of onset, duration, and severity, and all the information is recorded on a case report form.

### Withdrawals

The patients are free to withdraw from the trial upon request at any time and without providing reasons for their decision. Moreover, the primary investigator can withdraw study patients if continuation of the trial would be detrimental to the patient’s well-being. Withdrawals are documented on a case report form and in the patient’s medical records, and all ongoing severe adverse events are followed-up.

### Sample size

The sample size calculation was based on the primary endpoint and primary analysis for the intention-to-treat population. The sample size was calculated using the study’s primary objective- to confirm a 20% relative difference in myocardial glucose consumption uptake between olmesartan and valsartan treatment with a power of 90%. Based on previous reports, we assumed the myocardial glucose consumption value to be approximately 50 μl/g/min in DCMP patients [29, 30]. We expected that the relative difference in glucose uptake measurement would be at least 20% in DCMP patients. We adjusted the sample size, considering an estimated follow-up loss rate of 20% with a two-sided level of significance *α* = 5% and a power of 1-β = 90%, resulting in 20 patients in each group to show a difference with a two-sided Student’s *t*-test. The sample size was increased to up to 22 patients in each group with an expected drop-outs rate of 10% at the end of the study. Therefore, a total of 44 patients will be randomized and included in this study.

### Statistical analysis

Statistical analyses are performed on an intention-to-treat basis. The continuous variables are presented as mean ± SD or median and interquartile range and compared by Student’s *t*-test or Mann-Whitney *U* test, respectively, depending on the normality of the variables. The chi-squared test is used to analyze the categorical variables. Repeated measures between baseline and follow-up are evaluated using the paired *t*-test for continuous Gaussian-distributed parameters or the Wilcoxon test, as appropriate. Receiver operating curve analysis is used to assess the efficacy of using myocardial glucose consumption to predict clinical events. Spearman’s correlation analysis is used to analyze the correlations between myocardial glucose consumption and possible influencing factors. The independent influencing factors of myocardial glucose consumption are analyzed by multivariate logistic regression, and the regression coefficients, odds ratio, and 95% confidence intervals are calculated.

The safety analysis will include the calculation of frequencies and rates of adverse events reported in the two groups. This analysis will be performed on the full analysis set, which consists of patients who receive at least one dose of the study medication. Graphical methods, including scatter plots and boxplots, will be used to visualize the findings of the trial. Given that dropouts are expected, multiple imputations, based on regression methods, will be performed to complete the data analysis. A fully specified statistical analysis plan will be written before unmasking of the data. All statistical analyses will be performed using SPSS Version 16.0 or higher. Statistical significance is considered for *p*-values of < 0.05.

The report presenting the primary findings of this study will follow the Consolidated Standards of Reporting Trials 2010 guidelines. Study results will be disseminated to researchers and clinicians via publications and conference presentations. Authorship of published papers will follow established guidelines for defining the level of contribution that warrants authorship.

### Data management

This study will be coordinated by Dong-A University Hospital, which is responsible for the data processing and quality control. Completed electronic case report forms will be entered by trained site personnel and will be transmitted to a central data repository at Dong-A University Hospital. At regular intervals, all data will be transferred from electronic case report form to SPSS for statistical summarization, data description, and data analysis. Further cross-checking of the data will be performed in SPSS. Site qualification will be issued by Dong-A University Hospital for each modality before subject enrollment. Before beginning enrollment, eligible sites will be qualified by the imaging Core Laboratories based on-site surveys and on the successful transfer of one or more complete datasets with sufficient image quality and completeness for each modality. During the study, technical quality assessment of image and test acquisition will be accomplished on all studies by central repository research technicians trained by the imaging Core Laboratories. This ongoing review will ensure adequate quality and completeness of datasets. The collected data will be stored for 5 years after the end of the study and will be destroyed after that.

### Data monitoring

Data monitoring will be done semiannually to ensure that the electronic case report form is completed accurately. Data quality control will be regularly undertaken at each investigating site to ensure that the electronic case report form is accurately completed.

### Frequency and plans for auditing trial conduct

Auditing will be conducted by the Dong-A University Hospital auditing department.

### Protocol amendments

Any protocol modifications must be approved by the IRB prior to the implementation in this trial. Substantial changes will be added on ClinicalTrials.gov if there are protocol amendments from the original approved version.

### Plan for data assessment

All paper files are to be stored in locked file cabinets, and electronic files are to be stored in password-protected, encrypted files. Both paper and electronic files will be accessed by members of the research team only. The final dataset will reside with the principal investigator.

### Ancillary and post-trial care

Ancillary study is not planned. After the study, patients will receive a standard therapy.

## Discussion

### Renin-angiotensin-aldosterone system in DCMP

Overactivity of the RAAS is associated with poor patient prognosis in DCMP [7, 8]. Angiotensin II binds the angiotensin II receptor in targeted tissues and stimulates vasoconstriction and secretion of aldosterone, a steroid hormone, which mediates sodium reabsorption and water retention [31]. Aldosterone has an avid, renal sodium-water retaining effect and is considered a key hormone in the development of HF through its pleiotropic actions mediated by mineralocorticoid receptors [31]. Previous studies showed that chronic angiotensin II receptor stimulation-induced cardiac systolic dysfunction and electrical remodeling in the absence of hypertension [32, 33]. Further, a recent animal study reported that angiotensin II overstimulation led to an increased susceptibility to DCMP [34].

### Myocardial metabolism in DCMP

Patients with DCMP exhibit myocardial metabolism dysfunction characterized by decreased fatty acid metabolism and increased myocardial glucose metabolism [21, 22]. Recent studies suggested that RAAS might adversely affect cardiac energy metabolism in heart failure [5, 6]. Cardiac energy metabolic changes in heart failure can manifest as both a deficit in energy production by the heart, as well as a decrease in cardiac efficiency [35, 36]. Several investigators have shown a link between myocardial metabolism and impaired heart function. Myocardial energy efficiency in HF declines to as low as 15%, whereas in healthy volunteers or patients with coronary artery disease without HF, it is as high as 40% [37]. Alterations in myocardial substrate metabolism have been implicated in the pathogenesis of contractile dysfunction and HF [38]. Therefore, a further understanding of the relationship between the RAAS and myocardial metabolism could improve the control of HF and may lead to the development of new HF therapies.

### OVOID study (a comparison study of Olmesartan and Valsartan On myocardial metabolism In patients with Dilated cardiomyopathy)

Angiotensin blockade is a major DCMP therapeutic strategy; it provides a balanced reduction in preload and afterload and inhibits cardiac hypertrophy and remodeling, therefore reducing mortality and morbidity [19, 39, 40]. This trial is conducted to test the hypothesis that olmesartan could improve myocardial metabolism more effectively than valsartan for DCMP patients during a 6-month follow-up period. This is a short-term clinical study. However, this is the first trial investigating the potential benefits of 6 months olmesartan treatment on myocardial metabolism in DCMP patients. There are some pharmacological differences between the different ARBs. Among them, individual studies have demonstrated differences between ARBs efficacy in lowering blood pressure [13, 14, 41, 42]. However, as of March 2020, there have been no registered randomized trials comparing ARBs in patients with DCMP. Olmesartan has a double-chained domain consisting of carboxyl and hydroxyl groups, which can strongly combine with the angiotensin II receptor [15, 16]. Recently, Ishiyama reported that olmesartan increased ACE2 expression in the remodeling heart after myocardial infarction, which theoretically could contribute to the beneficial effects of ARB by facilitating increased cardiac Ang-(1-7) formation [43]. Olmesartan can block the angiotensin II receptors more efficiently because angiotensin II does not increase and can also enhance the effect of bradykinin through the inhibition of kininase II. Moreover, the increased Ang-(1-7) may have vasodilatory and organ-protective effects, such as the inhibition of vascular remodeling and cardiac hypertrophy [44–46]. If the potential benefits of olmesartan on myocardial metabolism are proved, the results of the present study may demonstrate the differential effects of ARBs as metabolic therapy.

## Supplementary Information

**Additional file 1: Appendix A.** SPIRIT 2013 Checklist: Recommended items to address in a clinical trial protocol and related documents*. **Appendix B.** World Health Organization Trial Registration Data Set. **Appendix C.** Informed consent (only for Korean).

## Data Availability

Not applicable.

## Abbreviations

ACE: Angiotensin-converting enzyme
AHA: American Heart Association
ARB: Angiotensin II receptor blocker
DCMP: Dilated cardiomyopathy
FDG: ^18^F-fluoro-2-deoxyglucose
HF: Heart failure
HMR: Heart-to-mediastinum ratio
NT-proBNP: N-terminal pro-B-type natriuretic peptide
LC: Lumped constant
LV: Left ventricle
LVEDD: Left ventricular end-diastolic diameters
LVEF: Left ventricular ejection fraction
LVESD: Left ventricular end-systolic diameter
MIBG: ^123^I-metaiodobenzylguanidine
NYHA: New York Heart Association
OVOID: A comparison study of Olmesartan and Valsartan On myocardial metabolism In patients with Dilated cardiomyopathy
PCARDP: PMOD Cardiac PET modeling
PET: Positron emission tomography
PG: Plasma glucose level
RAAS: Renin-angiotensin-aldosterone systems
ROI: Region-of-interest
RV: Right ventricle
SPECT: Single-photon emission computed tomography
TAC: Time-activity-curve
VOI: Volume of interest
WR: Wash-out rate
