# Supplementary material for: Design and rationale for a comparison study of Olmesartan and Valsartan On myocardial metabolism In patients with Dilated cardiomyopathy (OVOID) trial: study protocol for a randomized controlled trial
Source: Trials. 2022 Jan 15;23:36. doi: 10.1186/s13063-021-05970-7 (PMC8760768; doi:10.1186/s13063-021-05970-7)
Supplement: Supplementary file 1 — Additional file 1: Appendix A. SPIRIT 2013 Checklist: Recommended items to address in a clinical trial protocol and related documents*. Appendix B. World Health Organization Trial Registration Data Set. Appendix C. Informed consent (only for Korean). [file 13063_2021_5970_MOESM1_ESM.doc]

**Appendix A: SPIRIT 2013 Checklist: Recommended items to address in a clinical trial protocol and related documents***


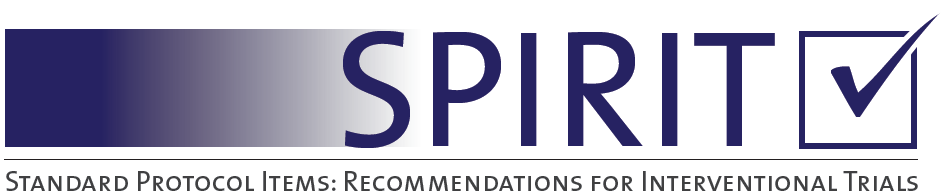


| Section/item | Item No | Description | Addressed on page number |
| --- | --- | --- | --- |
| **Administrative information** | | |  |
| Title | 1 | Descriptive title identifying the study design, population, interventions, and, if applicable, trial acronym | 1 |
| Trial registration | 2a | Trial identifier and registry name. If not yet registered, name of intended registry | 3 |
|  | 2b | All items from the World Health Organization Trial Registration Data Set | Appendix B |
| Protocol version | 3 | Date and version identifier | 6 |
| Funding | 4 | Sources and types of financial, material, and other support | 17 |
| Roles and responsibilities | 5a | Names, affiliations, and roles of protocol contributors | 1, 17 |
|  | 5b | Name and contact information for the trial sponsor | 1 |
|  | 5c | Role of study sponsor and funders, if any, in study design; collection, management, analysis, and interpretation of data; writing of the report; and the decision to submit the report for publication, including whether they will have ultimate authority over any of these activities | 17 |
|  | 5d | Composition, roles, and responsibilities of the coordinating centre, steering committee, endpoint adjudication committee, data management team, and other individuals or groups overseeing the trial, if applicable (see Item 21a for data monitoring committee) | 13,14 |
| Introduction |  |  |  |
| Background and rationale | 6a | Description of research question and justification for undertaking the trial, including summary of relevant studies (published and unpublished) examining benefits and harms for each intervention | 4 |
|  | 6b | Explanation for choice of comparators | 7 |
| Objectives | 7 | Specific objectives or hypotheses | 4,6 |
| Trial design | 8 | Description of trial design including type of trial (eg, parallel group, crossover, factorial, single group), allocation ratio, and framework (eg, superiority, equivalence, noninferiority, exploratory) | 5 |
| Methods: Participants, interventions, and outcomes | | |  |
| Study setting | 9 | Description of study settings (eg, community clinic, academic hospital) and list of countries where data will be collected. Reference to where list of study sites can be obtained | 5 |
| Eligibility criteria | 10 | Inclusion and exclusion criteria for participants. If applicable, eligibility criteria for study centres and individuals who will perform the interventions (eg, surgeons, psychotherapists) | 4, 5, Table 1 |
| Interventions | 11a | Interventions for each group with sufficient detail to allow replication, including how and when they will be administered | 7 |
|  | 11b | Criteria for discontinuing or modifying allocated interventions for a given trial participant (eg, drug dose change in response to harms, participant request, or improving/worsening disease) | 7,8 |
|  | 11c | Strategies to improve adherence to intervention protocols, and any procedures for monitoring adherence (eg, drug tablet return, laboratory tests) | 8 |
|  | 11d | Relevant concomitant care and interventions that are permitted or prohibited during the trial | 5 |
| Outcomes | 12 | Primary, secondary, and other outcomes, including the specific measurement variable (eg, systolic blood pressure), analysis metric (eg, change from baseline, final value, time to event), method of aggregation (eg, median, proportion), and time point for each outcome. Explanation of the clinical relevance of chosen efficacy and harm outcomes is strongly recommended | 7 |
| Participant timeline | 13 | Time schedule of enrolment, interventions (including any run-ins and washouts), assessments, and visits for participants. A schematic diagram is highly recommended (see Figure) | 5,6, Figure 1 |
| Sample size | 14 | Estimated number of participants needed to achieve study objectives and how it was determined, including clinical and statistical assumptions supporting any sample size calculations | 12 |
| Recruitment | 15 | Strategies for achieving adequate participant enrolment to reach target sample size | 8 |

| **Methods: Assignment of interventions (for controlled trials)** | | |  |
| --- | --- | --- | --- |
| Allocation: |  |  |  |
| Sequence generation | 16a | Method of generating the allocation sequence (eg, computer-generated random numbers), and list of any factors for stratification. To reduce predictability of a random sequence, details of any planned restriction (eg, blocking) should be provided in a separate document that is unavailable to those who enrol participants or assign interventions | 8 |
| Allocation concealment mechanism | 16b | Mechanism of implementing the allocation sequence (eg, central telephone; sequentially numbered, opaque, sealed envelopes), describing any steps to conceal the sequence until interventions are assigned | 8 |
| Implementation | 16c | Who will generate the allocation sequence, who will enrol participants, and who will assign participants to interventions | 8 |
| Blinding (masking) | 17a | Who will be blinded after assignment to interventions (eg, trial participants, care providers, outcome assessors, data analysts), and how | 8-10 |
|  | 17b | If blinded, circumstances under which unblinding is permissible, and procedure for revealing a participant’s allocated intervention during the trial | 6 |
| **Methods: Data collection, management, and analysis** | | |  |
| Data collection methods | 18a | Plans for assessment and collection of outcome, baseline, and other trial data, including any related processes to promote data quality (eg, duplicate measurements, training of assessors) and a description of study instruments (eg, questionnaires, laboratory tests) along with their reliability and validity, if known. Reference to where data collection forms can be found, if not in the protocol | 13 |
|  | 18b | Plans to promote participant retention and complete follow-up, including list of any outcome data to be collected for participants who discontinue or deviate from intervention protocols | 8 |
| Data management | 19 | Plans for data entry, coding, security, and storage, including any related processes to promote data quality (eg, double data entry; range checks for data values). Reference to where details of data management procedures can be found, if not in the protocol | 13,14 |
| Statistical methods | 20a | Statistical methods for analysing primary and secondary outcomes. Reference to where other details of the statistical analysis plan can be found, if not in the protocol | 12,13 |
|  | 20b | Methods for any additional analyses (eg, subgroup and adjusted analyses) | 13 |
|  | 20c | Definition of analysis population relating to protocol non-adherence (eg, as randomised analysis), and any statistical methods to handle missing data (eg, multiple imputation) | 13 |
| **Methods: Monitoring** | | |  |
| Data monitoring | 21a | Composition of data monitoring committee (DMC); summary of its role and reporting structure; statement of whether it is independent from the sponsor and competing interests; and reference to where further details about its charter can be found, if not in the protocol. Alternatively, an explanation of why a DMC is not needed | 13 |
|  | 21b | Description of any interim analyses and stopping guidelines, including who will have access to these interim results and make the final decision to terminate the trial | 14 |
| Harms | 22 | Plans for collecting, assessing, reporting, and managing solicited and spontaneously reported adverse events and other unintended effects of trial interventions or trial conduct | 13,14 |
| Auditing | 23 | Frequency and procedures for auditing trial conduct, if any, and whether the process will be independent from investigators and the sponsor | 14 |
| Ethics and dissemination | | |  |
| Research ethics approval | 24 | Plans for seeking research ethics committee/institutional review board (REC/IRB) approval | 6 |
| Protocol amendments | 25 | Plans for communicating important protocol modifications (eg, changes to eligibility criteria, outcomes, analyses) to relevant parties (eg, investigators, REC/IRBs, trial participants, trial registries, journals, regulators) | NA as no plan for communicating important protocol modifications. |
| Consent or assent | 26a | Who will obtain informed consent or assent from potential trial participants or authorised surrogates, and how (see Item 32) | 6 |
|  | 26b | Additional consent provisions for collection and use of participant data and biological specimens in ancillary studies, if applicable | NA as no use of participant data and biological specimens are collected as part of this trial. |
| Confidentiality | 27 | How personal information about potential and enrolled participants will be collected, shared, and maintained in order to protect confidentiality before, during, and after the trial | 14 |
| Declaration of interests | 28 | Financial and other competing interests for principal investigators for the overall trial and each study site | 17 |
| Access to data | 29 | Statement of who will have access to the final trial dataset, and disclosure of contractual agreements that limit such access for investigators | 14 |
| Ancillary and post-trial care | 30 | Provisions, if any, for ancillary and post-trial care, and for compensation to those who suffer harm from trial participation | 6 |
| Dissemination policy | 31a | Plans for investigators and sponsor to communicate trial results to participants, healthcare professionals, the public, and other relevant groups (eg, via publication, reporting in results databases, or other data sharing arrangements), including any publication restrictions | 13 |
|  | 31b | Authorship eligibility guidelines and any intended use of professional writers | 13 |
|  | 31c | Plans, if any, for granting public access to the full protocol, participant-level dataset, and statistical code | NA as no plan for granting public access to the full protocol, participant-level dataset, and statistical code. |
| Appendices |  |  |  |
| Informed consent materials | 32 | Model consent form and other related documentation given to participants and authorised surrogates | Appendix C |
| Biological specimens | 33 | Plans for collection, laboratory evaluation, and storage of biological specimens for genetic or molecular analysis in the current trial and for future use in ancillary studies, if applicable | NA as no biological specimens are collected as part of this trial. |

*It is strongly recommended that this checklist be read in conjunction with the SPIRIT 2013 Explanation & Elaboration for important clarification on the items. Amendments to the protocol should be tracked and dated. The SPIRIT checklist is copyrighted by the SPIRIT Group under the Creative Commons “[Attribution-NonCommercial-NoDerivs 3.0 Unported](http://www.creativecommons.org/licenses/by-nc-nd/3.0/)” licens

**Appendix B: World Health Organization Trial Registration Data Set**

1. **Primary Registry and Trial Identifying Number:** ClinicalTrials.gov; NCT04174456; URL https://clinicaltrials.gov/ct2/show/ NCT04174456.
2. **Date of Registration in Primary Registry:** November 18, 2019
3. **Secondary Identifying Numbers:** NA.
4. **Source(s) of Monetary or Material Support:** Daewoong Pharmaceutical, 12, Bongeunsa-ro 114 gil, Gangnam-gu, Seoul, Republic of Korea; Phone +825508800
5. **Primary Sponsor:** Dong-A University hospital, 1 Dongdae-sin-dong 3-ga, Seo-gu, Busan 602-715, Korea; Phone +82512402059; Fax: +82512402044; e-mail: cardiopark@gmail.com
6. **Secondary Sponsor(s):** NA.
7. **Contact for Public Queries:** Sua Jo, MD, Division of Cardiology, Department of Internal Medicine, Dong-A University College of Medicine, 1 Dongdae-sin-dong 3-ga, Seo-gu, Busan 602-715, Korea; Phone: +82-512402059; Fax: +82-512402044; e-mail: flysofina@gmail.com
8. **Contact for Scientific Queries:** Kyungil Park, MD, PhD Associate Professor, Division of Cardiology, Department of Internal Medicine, Dong-A University College of Medicine, 1 Dongdae-sin-dong 3-ga, Seo-gu, Busan 602-715, Korea; Phone: +82-512402059; Fax: +82-512402044; E-mail: cardiopark@gmail.com
9. **Public Title:** Comparison study of Olmesartan and Valsartan On myocardial metabolism In patients with Dilated cardiomyopathy
10. **Scientific Title:** Design and rationale for a comparison study of Olmesartan and Valsartan On myocardial metabolism In patients with Dilated cardiomyopathy (OVOID) trial
11. **Countries of Recruitment:** Republic of Korea.
12. **Health Condition(s) or Problem(s) Studied:** Dilated cardiomyopathy.
13. **Intervention:**
14. Intervention arm

- Name: Treatment with Olmesartan.
- Description: Olmesartan treatment (20 mg once daily)

1. Control arm: Treatment with Valsartan.
2. **Key Inclusion and Exclusion Criteria**
3. Inclusion criteria: Diagnosis of dilated cardiomyopathy. Additional requirements are New York Heart Association (NYHA) functional classes of III and IV.
4. Exclusion criteria: less than 20 years or more than 85 years old, the presence of hemodynamic instability, known intolerance to olmesartan and valsartan, coronary artery disease based on coronary angiography (≥ 50% stenosis in ≥ 1 of the major coronary arteries) and/or a history of myocardial infarction or angina pectoris, acute or subacute stage of myocarditis, primary valve disease, excessive use of alcohol, expected or performed cardiac resynchronization therapy and heart transplantation, stress-provoked Takotsubo cardiomyopathy, tachycardia‐induced cardiomyopathy, peripartum cardiomyopathy, Cor pulmonale, impaired renal function (estimated glomerular filtration rate of < 60 ml/min/1.73m^2^, a life expectancy of less than one year, and an the inability to provide informed consent.
5. **Study Type**
   1. Type of study: interventional.
   2. Study design:
      - Method of allocation: randomized
      - Masking: no
      - Assignment: parallel
      - Purpose: comparing the myocardial metabolism of olmesartan vs. valsartan in nonischemic myocardial segments of patients with dilated cardiomyopathy.
6. Phase: NA
7. Allocation concealment mechanism and sequence generation: Eligible patients are randomly assigned in a 1:1 ratio to receive olmesartan or valsartan. Random treatment assignments will be generated using Excel spreadsheet software.
8. **Date of First Enrollment:** December 20, 2019.
9. **Target Sample Size:** 40
10. **Recruitment Status: recruiting.**
11. **Primary Outcome(s)**
    - Name: Myocardial metabolism
    - Method of measurement: Myocardial glucose consumption measured by ^18^F-fluoro-2-deoxyglucose positron emission tomography
    - Time points: 6 months.
12. **Key Secondary Outcomes**
    1. Name: Changes in myocardial glucose consumption, NT-proBNP levels, left ventricular ejection fraction, NYHA functional class from baseline to the last available observation after treatment, and the occurrence of predefined clinical events after receiving the study agent

**Appendix C: Informed consent (only for Korean)**

**피험자용 설명 및 동의서**

**연구 과제명**: 확장성 심근병증 환자에서 올로스타 약제의 심근 생존량에 대한 연구

**연구 책임자명**: 동아대학교 병원 순환기내과 교수 박 경 일

1. **본 임상시험은 연구목적으로 수행됩니다.**

본 임상연구의 목적은 확장성 심근병증 환자의 치료에서 올로스타의 투여가 발사르탄과로슈바스타틴 복합요법과 비교해서 심근 생존량의 차이가 있는가를 알아보고자 하는 비교 연구입니다.

1. **연구방법 및 예측 효능, 효과**

확장성 심근병증은 심장 근육의 이상으로 인해 심장이 확장되고 심장기능이 저하되는 심장질환이다. 확장성 심근병증은 수축기 기능장애성 심부전을 야기하는 질병입니다.

확장성 심근병증은 기본적으로 심부전 관련 증상이 나타나기 때문에, 치료에 사용되는 약물은 심부전 약제와 동일합니다. 심부전 약물로도 증상이 완화되지 않거나 상태가 악화될 경우에는 심장이식이 필요할 수도 있습니다. 확장성 심근병증은 제대로 치료를 하지 않을 경우 5년이내 사망률이 70% 정도이며, 사망자의 절반은 부정맥으로 급사하는 것으로 알려져 있으며, 확장성 심근병증의 가장 중요한 치료는 약물 요법입니다.

확장성 심근병증이 개선되기 위해서는 심장의 근육(심근)으로 혈액 공급이 잘되어야 하는데, 그동안 심근의 혈류량을 정확하게 측정할 수 있는 장비가 없었습니다.

최근에 심근 생존량을 정확하게 측정할 수 있는 장비가 개발되었습니다. 이것은 당을 이용한 심장 양전자 단층촬영 검사방법으로, 심근 생존량을 평가하는 데 있어 신뢰성이 높다고 인정되는 기법이고 침습적이지 않으므로, 본 연구에서 안전하게 심근 생존량을 평가할 수 있습니다.

올로스타는 최근에 출시된 고혈압 및 고지혈증 치료 복합제로 혈압 치료와 고지혈증 치료를 동시에 할 수 있도록 개발된 약제입니다. 올로스타는 안지오덴신 수용체와 결합을 억제하는 약제인 올메르사탄과 고지혈증을 치료하는 로슈바스타틴의 복합제입니다. 올메르사탄은 심부전 환자의 치료약제로 사용되고 있으며, 기존의 안지오텐신 수용체의 결합억제 약물과 비교해서 안지오텐신 수용체의 결합 억제 능력이 가장 강력하다고 알려져 있습니다. 로슈바스타틴은 혈관 내피세포 기능 개선에 도움이 될 수 있다는 연구 결과가 보고되어 왔고, 이런 기전 등을 통해 심근 생존량 개선에 영향을 미칠 수 있다는 의견들이 여러 연구자 등에 의해 보고된 바가 있습니다. 그러나 심근 생존량을 정확하게 평가하지 못했기 때문에 약제가 심근 생존량에 미치는 영향을 분석할 수 없었습니다.

이에 본 연구에서는 확장성 심근병증 환자에서 올로스타 투여가 기존 약제와 비교해서 심근 생존량에 미치는 영향에 대해 규명해 보고자 합니다.

1. **본 임상시험의 선정기준**

호흡곤란으로 동아대학교 의료원을 방문해서 확장성 심근병증으로 진단된 후에 약물 요법이 필요한 환자가 본 연구에 참여할 수 있습니다.

1. **시험에 사용되는 약물**

본 연구에서 사용되는 올로스타와 발사르탄 및 로슈바스타틴은 이미 국내에서 널리 사용되어 온 약제이며 효과와 안전성이 확립되어 심부전 및 고지혈증의 치료에 있어 필수적으로 사용을 권장하고 있는 약품입니다. 올로스타를 구성하는 약제는 올메르사탄이고, 발사르탄과 더불어 심부전의 예후를 개선시키는 약제로 알려져 있습니다.

올로스타를 구성하는 또다른 약제는 로슈바스타틴으로 고지혈증의 치료제로 알려져 있으나 이외에 혈관 내피세포 기능을 개선시키고 관상동맥내 혈류량을 개선시킬 수 있다고 알려져 있어 고지혈증 치료 목적 이외에 관상동맥질환의 예후 개선 치료로서 매우 중요한 약제입니다.

1. **본 연구에 사용되는 기구**

본 연구에서 새로이 사용되는 기구는 없습니다.

1. **본 연구에 참여하게 됨으로써 받게 되는 검사 및 절차**

본 연구에 참여하시게 됨으로써 추가적으로 심장양전자단층촬영 검사를 받게 됩니다. 이 외 임상 연구에 참여하지 않는 다른 환자분들과 같은 방법으로 심장초음파 검사화 혈액 검사가 시행될 예정이며 검사 절차도 임상 연구에 참여하지 않는 다른 환자들과 똑같습니다. 퇴원 후, 외래 추적 관찰 경과 및 약물 치료도 같습니다.

1. **본 연구를 위해서 피험자가 준수해야 하는 사항**

본 연구에 참여하시는 환자분이 준수해야 하는 사항은 임상연구에 참여하지 환자와 같습니다.

1. **본 임상시험의 검증되지 않은 실험적인 측면**

해당 사항 없습니다.

1. **임상시험에 참여함으로써 피험자에게 미칠 것으로 예견되는 위험(부작용)이나 불편사항**

해당 사항 없습니다.

1. **이 임상시험에 참여함으로써 기대되는 이익**

참여 환자를 대상으로 심장 양전자 단층촬영 검사가 시행되는데 해당 검사를 무상으로 제공받게 됩니다.

1. **본 질환으로 선택할 수 있는 다른 치료방법 및 이러한 치료의 잠재적 위험과 이익**

확장성 심근병증의 약물 치료는 임상 시험에 참여하지 않는 환자분들과 동일하며, 약제의 부작용에 대한 검사 및 평가는 동일하게 수행하게 됩니다. 따라서, 본 질환으로 선택할 수 있는 특별한 다른 치료방법이 없으므로 본 연구에 따른 잠재적 위험은 예상되지 않습니다.

1. **예상 참여기간 및 본 시험에 참여하는 대략의 전체 피험자수**

확장성 심근병증의 진단 후 약물 요법 시행해서 6개월까지이며, 40명이 이 연구에 참여하게 됩니다. 40명의 참여자는 무작위 배정에 의해 20명은 올로스타를 복용하고, 20명은 발사르탄과 로슈바스타틴을 복용하게 됩니다.

1. **임상시험과 관련된 손상이 발생하였을 경우 피험자에게 주어질 보상이나 치료 방법**

예상치 못한 이상 반응이 발생할 경우, 신속하게 해당 분야의 전문의에게 진료를 받도록 하여 조기에 이상 반응이 치료될 수 있게 하며 환자의 부담이 최소화되도록 노력할 것입니다. 치료 도중에 발생하는 어떠한 종류의 부작용에 대하여도 세심하게 관찰이 기울여질 것이며 부작용이 발생하면 본 의료진 전원에게 알려지고, 가장 좋은 치료가 수행되도록 할 것입니다. 따라서 치료 도중이나 후 어떠한 이상이라도 발견되면 의료진에게 언제든지 문의하시기 바랍니다.

1. **임상시험에 참여함으로써 받게 되는 금전적 보상의 여부 및 참여 정도에 따른 조정 정도 또는 임상시험에 참여함으로써 피험자에게 추가적으로 발생이 예상되는 비용**

본 연구에 참여하시더라도 경제적인 보상은 없습니다. 또한 본 연구에 참여하시더라도 추가적으로 발생되는 비용은 없습니다.

1. **다음의 경우에는 보상하지 않습니다.**

1) 연구책임자의 후원 하에 시행되지 않았거나 연구자가 제공하지 않은 의약품 등으로 인하여 발생한 이상반응의 경우

2) 서로 합의한 임상시험계획서를 준수하지 않아 일어난 손상(임상시험 계획서에 요구되지 않은 검사 또는 치료적 조치로 인한 경우 포함)

3) 피험자의 부주의로부터 발생된 경우

4) 환자의 기저 질환의 진행 및 악화에 의해 발생한 경우

5) 심부전 치료 중 발생할 수 있는 일반적인 부작용

6) 질병의 자연 경과에 의해 발생한 손상

1. **본 임상연구에서 기타 고려사항은 다음과 같습니다.**

**피험자가 받게 되는 새로운 정보**: 임상연구 지속 참여 의지에 영향을 줄 수 있는 새로운 정보가 얻어지면 적시에 본인 또는 대리인에게 알려집니다. 본 연구진행 중 본인에게 영향을 줄 수도 있는 새로운 정보를 연구자가 획득하게 되면 그 내용을 통보 받을 수 있습니다.

**임상연구 참여의 제한**: 연구책임자가 필요하다고 판단 될 경우 본인의 동의 없이도 본 연구 참여에서 제한될 수 있습니다. 또한, 이 경우 연구책임자 또는 동의서상에 명시된 연구자에게 통보 함으로써 본 연구에 불참할 수 있습니다.

**자유의사에 따른 임상연구에의 참여**: 환자분이 이 임상연구에 참여해야 할 의무는 없으며, 참여 여부는 본인 자유의사에 의하여 결정됩니다. 동의서에 서명하고 임상연구에 참여한 후에도 언제나 환자분이 원하면 이를 중단할 수 있습니다. 그렇더라도 본 병원에서 계속 치료 받는데 있어서 불이익이나 다른 환자와의 차별은 일체 없습니다.

**비밀 보장**: 모니터요원, 점검을 실시하는 자, 심사위원회 및 식품의약품안전청장은 피험자의 비밀보장을 침해하지 않고 관련규정이 정하는 범위 안에서 임상연구의 실시절차와 자료의 신뢰성을 검증하기 위해 본인의 의무기록을 직접 열람할 수 있습니다. 본 동의서에 서명함은 이러한 자료의 직접 열람을 허용한다는 것을 의미합니다. 본 임상연구의 결과가 출판될 경우 피험자의 신원은 비밀 상태로 유지됩니다.

본 연구에 대해 질문이 있거나 연구 중간에 문제가 생길 시 다음 연구 담당자에게 연락하십시오.

이름: 고경미 전화번호 051-240-5740

만일 어느 때라도 피험자로서 귀하의 권리에 대한 질문이 있다면 다음의 동아대학교 병원 임상연구심의위원회로 연락하십시오.

동아대학교 병원 임상연구심의위원회 전화번호: 051-240-2611 (동아대학교병원)

**동 의 서**

1. 나는 이 설명서를 읽었으며 담당 연구원과 이에 대하여 의논하였습니다.
2. 나는 위험과 이득에 관하여 들었으며 나의 질문에 만족할 만한 답변을 얻었습니다.
3. 나는 이 연구에서 얻어진 나에 대한 정보를 현행 법률과 임상연구심의위원회 규정이 허용하는 범위 내에서 연구자가 수집하고 처리하는데 동의합니다.
4. 나는 이 연구에 참여하는 것에 대하여 자발적으로 동의합니다.
5. 나는 담당 연구자나 위임 받은 대리인이 연구를 진행하거나 결과 관리를 하는 경우와 보건 당국, 학교 당국 및 동아대학교 병원 임상연구심의위원회가 실태 조사를 하는 경우에는 비밀로 유지되는 나의 개인 신상 정보를 직접적으로 열람하는 것에 동의합니다.
6. 나는 언제라도 이 연구의 참여를 철회할 수 있고 이러한 결정이 나에게 어떠한 해도 되지 않을 것이라는 것을 압니다.
7. 나의 서명은 이 동의서의 사본을 받았다는 것을 뜻하며 연구 참여가 끝날 때까지 사본을 보관하겠습니다.

| 피험자 성명 |  | 서명 |  | 날짜 (년/월/일) |  |
| --- | --- | --- | --- | --- | --- |
| 동의서 받은 연구원 성명 |  | 서명 |  | 날짜 (년/월/일) |  |
| 연구책임자 성명 |  | 서명 |  | 날짜 (년/월/일) |  |
| 만일 있을 경우 | | | | | |
| 법적 대리인 성명 |  | 서명 |  | 날짜 (년/월/일) |  |
| 입회인 성명 |  | 서명 |  | 날짜 (년/월/일) |  |
